# A Guide to Selecting Participatory Research Methods Based on Project and Partnership Goals

**DOI:** 10.35844/001c.32605

**Published:** 2022-05-23

**Authors:** Stephanie R. Duea, Emily B. Zimmerman, Lisa M. Vaughn, Sónia Dias, Janet Harris

**Affiliations:** 1 School of Nursing, University of North Carolina at Wilmington; 2 Department of Family Medicine and Population Health, Division of Epidemiology, VCU Center on Society and Health, Virginia Commonwealth University; 3 College of Medicine, Cincinnati Children’s Hospital Medical Center/University of Cincinnati; 4 Public Health Research Center, NOVA National School of Public Health; 5 School of Health & Related Research, University of Sheffield

**Keywords:** stakeholder engagement, community-engaged research, participatory health research, research methods

## Abstract

Participatory research engages community stakeholders in the research process, from problem identification and developing the research question, to dissemination of results. There is increasing recognition in the field of health research that community-engaged methods can be used throughout the research process. The volume of guidance for engaging communities and conducting participatory research has grown steadily in the past 40+ years, in many countries and contexts. Further, some institutions now require stakeholder engagement in research as a condition of funding. Interest in collaborating in the research process is also growing among patients and the public. This article provides an overview for selecting participatory research methods based on project and partnerships goals.

Participatory research engages community stakeholders to work alongside academics across all stages of the research process, from problem identification and developing the research question to the dissemination of results. Community and stakeholder engagement can be defined as the involvement of relevant stakeholders as full partners in all phases of research, requiring relationships built on trust and respect regardless of partners’ training or experience in science or research (Woolf et al., 2016). In this context, and throughout the research process, participation is the defining principle “recognizing the value of each person’s contribution to the co-creation of knowledge in a process that is not only practical, but also collaborative and empowering” (ICPHR, 2013, p. 5). Cornwall and Jewkes (1995) compare participatory and conventional research processes and note that “the key difference between participatory and other research methodologies lies in the location of power in the various stages of the research process.” Authentic engagement in the research process develops community capacity to be co-producers of the research process and outcomes.

Participatory Health Research (PHR) is a research paradigm whereby the research process, in its entirety, is a partnership between stakeholders with different backgrounds and perspectives, such as researchers, professionals, community members, policy makers, and others (ICPHR, 2013). The collaborative nature of participatory research necessitates a trustful relationship between the researchers and community partners, which in turn can promote the community’s acceptance of the study. Community acceptance may improve participation, data quality, and uptake of results (Abma et al., 2019; Balazs & Morello-Frosch, 2013; Ramsden et al., 2010).

The use of a participatory research approach enables the integration of stakeholder perspectives and research on questions prioritized by communities that are often not considered by researchers. Hence, the engagement of communities in the study design contributes to production of data that are more adequate and relevant for them (Balazs & Morello-Frosch, 2013; Vaughn & Jacquez, 2020). This engagement is also valuable in the development and validation of data collection instruments, development of tailored recruitment approaches, and data collection (Vaughn & Jacquez, 2020). By having a deep knowledge of the community context, community partners can help researchers identify locations and social networks to facilitate participant recruitment and data collection. This is particularly true with underrepresented populations as these groups might be more reluctant to participate in research studies (Dias et al., 2018; Rodriguez Espinosa & Verney, 2020). Additionally, the participatory research process contributes to the promotion of capacity building, empowerment of communities to address their health needs and priorities, and an increased sense of ownership of the project (Dias & Gama, 2014; Israel et al., 2010). Finally, the participatory processes can stimulate the receptiveness of communities to policies and recommendations that arise from the research results—but engaging with policymakers can also be a key strategy for translating research into policy development and implementation (Ogbe et al., 2018).

There is increasing recognition in the field of health research that community-engaged methods can be used throughout the research process (e.g., when developing the research question, designing and conducting the study, dissemination of findings) (Vaughn & Jacquez, 2020). The volume of guidance documents for engaging communities and conducting participatory research has grown steadily in the past 40+ years. This trend has occurred in many countries and contexts. Further, some institutions now require stakeholder engagement as a condition of funding. Interest in collaborating in the research process is also growing among patients and the public (Rutten et al., 2015). To move in this direction, both researchers and communities need to develop the capacity for conducting participatory research and need to be able to identify useful and appropriate methods suited to their partnerships, project goals, and processes.

PHR has integrated a wide range of existing research methods and approaches. While participatory research includes both quantitative and qualitative methods, these methods are often adapted for the participatory research process (ICPHR, 2013), which sometimes raises questions about the methodological rigor of PHR. These challenges have been met by the recognition that PHR can be judged by its “adherence to validity criteria specific to participatory research approaches: participatory, intersubjective, contextual, catalytic, ethical, and empathic validity” (ICPHR, 2013, pp. 19–20). Another important remedy is the use of methods for engaging stakeholders and co-producing research that have developed along with the growing interest in participatory research. These methods reflect PHR’s diverse goals and the wide range of contexts in which it is conducted.

The purpose of this article is to provide an overview of participatory research methods for researchers new to PHR and for experienced PHR researchers seeking information about methods they have not used before. The authors selected a diverse range of participatory research methods and provide examples of how they can be used for different research goals (see Tables 1–5). We derived our categories and examples from collective experience, the existing research methods literature, and through the iterative process described below. Our resulting categorization is not intended to provide a comprehensive catalog of methods. Further, we recognize that the selected categorization is just one way of organizing these methods and that in many, if not all, cases, these methods overlap and could be utilized in other ways and other phases of the research process.

## Methods

The International Collaboration for Participatory Health Research (ICPHR) was founded in 2009 and is a scientific, nongovernmental network and a community of practice focused in part on synthesizing the knowledge and experience of PHR in different countries, addressing issues of quality, credibility, and impact (ICPHR, 2020, p. 3). Members meet annually to discuss issues of common concern and convene workgroups on specific topics and projects. This work originated at the ICPHR annual meeting in 2019. At that time, ICPHR members interested in PHR methods formed a working group on methods and discussed the need for sharing resources related to methods that were developed or adapted for participatory research and collaboration. The working group included faculty members from the United States, Portugal, and England whose work focused on implementing, advancing, and evaluating PHR methods. Having used and written about a wide variety of PHR methods (e.g., Dias et al., 2018; Dias & Gama, 2014; Harris, Booth, et al., 2018; Harris, Cook, et al., 2018; Harris et al., 2019; Jacquez et al., 2013; Smith et al., 2020; Vaughn et al., 2009, 2016; Vaughn & DeJonckheere, 2019; Vaughn & Jacquez, 2020; Vaughn & Lohmueller, 2014; Zimmerman, 2021; Zimmerman et al., 2020), the authors began the process of developing this article by creating an annotated list of methods they were aware of and creating a matrix to identify different characteristics of these methods. Next, we grouped the methods into the domains presented here and then conducted literature reviews within each domain to identify additional methods and relevant literature, including foundational articles and more recent implementation studies. For each method, we refer readers to a select list of articles that highlight the background of each method and implementation examples.

Our overview of participatory methods is organized in five domains: 1) Engagement and capacity building; 2) Exploration and visioning; 3) Visual and narrative; 4) Mobilization; and 5) Evaluation. Below, we define each domain and provide examples of methods within each. We describe the type of method, typical goals for which the method is employed, participants engaged, and some strengths and weaknesses that users of these methods have identified. This summary is meant to orient readers to the types of methods employed and their potential uses and does not aim to provide any definitive description or assessment. We also include citations that provide examples of how the different types of methods have been used and urge readers who are learning about these methods to seek out the depth of information needed for their purposes by further exploring the literature.

Categorization of the methods into the five domains was conceived as a way to link each method to a collaborative goal rather than a specific stage in the research process, reflecting the flexibility to be useful at various research stages. For example, the community consultation provided by a Community Engagement Studio may be useful during research development to refine questions, identify ethical issues, and identify partners, but may also be useful later in the research process to address dissemination and translation. Similarly, a Photovoice project may help identify community priorities at the beginning of research or may spur advocacy or policy initiatives at later stages.

### Domain: Engagement and Capacity Building

Engagement of stakeholders encompasses a spectrum of participation that ranges from one-off consultation and short-term intensive workshops to periodic engagement to ongoing and active collaboration and ownership of the research process. The establishment of partnerships and networks is more likely to build capacity for doing participatory research than one-off or short-term engagement. While engagement is often described as a process where academic researchers attempt to involve other stakeholders, in participatory research, communities may take the lead by identifying issues that require research and approaching academics and health practitioners to explore collaborations.

Researchers who are applying for funding often use one-off engagement at the research planning stage. At the planning stage, people with lived experience of a health condition may be asked to critique proposed research designs (Shippee et al., 2015). *Community engagement (CE) studios* are one approach where focused engagement is used to obtain feedback on existing ideas for research (Joosten et al., 2015, 2018). In a brief (usually two hour) CE Studio session, a skilled and neutral moderator facilitates discussion between stakeholders and researchers with the aim of promoting co-learning and obtaining project-specific input that increases the relevance and acceptability of the proposed research. The contributions are used to refine research design, modify patient information and patient consent, and inform recruitment processes and patient compensation (Shippee et al., 2015).

*CBPR charettes*, in contrast, aim for more intense engagement through facilitated, transdisciplinary workshops lasting for three to five days. Days may be split, or interspersed with small group work, to promote knowledge exchange and discourse on specific issues (Kennedy, 2017). In CBPR, charrettes have been used to convene participants from diverse fields and knowledge bases, with the aim of identifying issues that need to be addressed through research, assessing the feasibility of proposed projects, and developing a plan that can be used as the basis of a more detailed roadmap for a transdisciplinary research project (Samuel et al., 2018). These projects may be broader than clinical research, encompassing health and the environment, vulnerable groups, public health issues such as violence, community development and planning (Kennedy, 2017; Smith et al., 2020). Charettes use a specific objective to build intergroup collaboration, which in turn serves to build capacity for developing longer-term partnerships (Smith et al., 2020). They do this by leveraging participants’ expertise to set up Community Advisory Boards and establish governance and decision-making structures, while clarifying initial expectations and roles (Samuel et al., 2018). Consistent participation in the charette and communication of charette activities to a wider stakeholder group are important in terms of fostering longer-term engagement.

Over the longer term, *patient research networks (PRNs)* enhance research relevance and usefulness throughout a clinical research study by coordinating patient involvement at different project stages, including recruiting participants for studies, partnering with researchers to coordinate the collection and analysis of data, and developing strategies for disseminating results. The common aim across patient research networks is to ensure that patients are included as partners across all stages, including their position in leadership and decision-making roles (Marschhauser et al., 2021). One of the benefits of early engagement has been establishing what matters to patients when they take part in research (Natafgi et al., 2019). *Patient-powered research networks (PPRNs)* share the aims of other PRN models, but also strive to create a national data and engagement infrastructure across diverse United States health institutions (Marschhauser et al., 2021). PPRNs enlist patients to collect and share their data, primarily with academic researchers, in order to create large data sets. Individuals with one condition or a set of related conditions contribute self-reported data gathered via remote monitoring devices, or share data from their electronic health records, with the PPRN, which coordinates large-scale data collection for researchers (Fleurence et al., 2014).

*Community Advisory Boards (CABS)* aim to solidify partnerships through deeper participation and ongoing stakeholder contribution to and co-production of research (Dias et al., 2018; Pratt & Hyder, 2016). Successful partnerships engage people who are committed to reducing health disparities, for example, and who have reputations as doers and consensus makers (Horowitz et al., 2011). Incorporating regular discussion where members can express concerns and negotiate conflict is key (Rowe & Frewer, 2005), in addition to formal and informal training where different skills and expertise are shared. These activities can enable CAB members to recognise and value diverse opinions and skills while developing a shared purpose and solidarity, which further builds capacity and sustainability of the partnership (Harris et al., 2019).

### Domain: Exploration and Visioning

Participatory methods that we classified in the Exploration and Visioning domain support various stages of research development, such as community dialogue, stakeholder priority setting, developing research questions, and exploring the meaning, causes, or solutions to specific problems. All methods categorized in the Exploration and Visioning domain support a wide range of group involvement and are intended to benefit from relevant stakeholder representation across a group or system (e.g., patients, physicians, other healthcare providers, clinic administrators, staff, and family/community members for a research study about a chronic disease for which patients are seen in a specialty clinic).

An example of an Exploration and Visioning process method that can be used to support research development is the multi-stakeholder *SEED Method* (Zimmerman et al., 2020; Zimmerman & Cook, 2021), which brings in multiple groups of patient and stakeholder participants in the research team, by creating “topic groups” that build conceptual models and develop research questions or strategies, and also through consultation such as focus groups or interviews. The SEED Method has been used to develop and prioritize research questions on lung cancer disparities (Rafie et al., 2019), diet and behavioral management of diabetes and hypertension (Zimmerman et al., 2017), and to develop strategies for addressing the opioid crisis and working with stakeholders to develop and implement action plans (Zimmerman et al., 2020).

*Concept mapping methodology* is a mixed-methods research approach that integrates qualitative and quantitative data collection methods of brainstorming, card sorting, and ratings with the multivariate statistical techniques of multidimensional scaling and cluster analysis to create a data-driven visual representation of thoughts or ideas of a group (Kane & Trochim, 2007; Trochim, 1989). Concept mapping (Jackson & Trochim, 2002; Rosas & Kane, 2012) works well within a participatory framework because of the structured steps that include diverse perspectives of multiple constituencies within a group or community (Vaughn et al., 2017). Concept mapping has been used widely in PHR as a foundation for evidence-based action planning or program and policy development that can be co-created with stakeholders. For instance, Szaflarski and colleagues (2015) used concept mapping to explore HIV-related stigma in the Cincinnati Black Faith community, and Ahmad et al. (2012) used concept mapping methodology working with South Asian immigrant women to identify barriers to mammography and solutions. Methodological studies support the validity and utility of concept mapping (Jackson & Trochim, 2002; Rosas & Kane, 2012).

Many of the Exploration and Visioning methods are intended to engage large groups in a process where all stakeholders can participate and be heard, with an emphasis on action planning and areas of agreement/consensus (e.g., community forums, Future Search Conference, Group Level Assessment, World Café). For instance, *Future Search Conference (FSC)* is a large-group methodology that brings a whole system into the room to work on a task-focused agenda (Serrat, 2017; Weisbord & Janoff, 2000, 2010). FSCs have been used in a variety of settings with successful results, including enhanced participant involvement and awareness, confirmation of mutual values, and increased commitment to future action (Magnus et al., 2016). FSCs are most commonly used in organizational settings to facilitate collective strategic planning and visioning, leaving opportunities for wider usage in PHR.

The World Café invites stakeholders to engage in small group discussions by rotating tables where specific questions and ideas are discussed and then shared with the larger group. Conversations are encouraged through a social café style setting and the use of Appreciative Inquiry. Although conversations take place in small groups, a relatively large number of people can come together at one World Café event (Fouché & Light, 2011). MacFarlane et al. (2017) held World Cafés with community organizations, community participants, academics, clinicians, and health service planners/policy makers in Ireland to prioritize research questions for a primary health care research group. The two sessions engaged 63 participants in all. They also report on a series of five World Cafes held in the U.S. with refugee and immigrant communities, each with approximately 45 to 55 participants, to develop research questions on diabetes mellitus.

Another Exploration and Visioning method is *Group Level Assessment (GLA)*. Qualitative and participatory, GLA is intended for research and evaluation with large groups of stakeholders who have an equal voice in data generation, data analysis, and action planning (Vaughn & DeJonckheere, 2019; Vaughn & Lohmueller, 2014). GLA utilizes an action-based, collaborative research process and is popular across a wide variety of disciplines to assess and explore various topics. GLA proceeds through seven structured steps that vary in terms of individual, small group, and large group activities: 1) Climate Setting; 2) Generating; 3) Appreciating; 4) Reflecting; 5) Understanding; 6) Selecting; and 7) Action. GLA is useful across a variety of settings and allows diverse stakeholders to work together to identify, prioritize, and take action about issues of importance (K. E. Graham et al., 2015; Vaughn et al., 2011). In the health arena, examples where GLA has been used include factors influencing the use of physiological monitors for hospitalized children (Schondelmeyer et al., 2019); African American fathers’ perceptions of involvement in the pediatric medical home (Bignall et al., 2018); and communication when caring for children with limited English proficiency during inpatient hospital stays (Choe et al., 2019).

### Domain: Visual and Narrative

The Visual and Narrative domain includes participatory research methods that use visual and narrative approaches to data collection, analysis, and interpretation. The methods in this domain generally emphasize the sharing and co-production of stakeholder experiences and ideas through alternative, multimodal approaches to data collection and interpretation. Participatory visual and narrative methods are guided by stakeholder interests and priorities, “putting the methods literally in the hands of participants themselves and allowing for greater access to social research knowledge beyond the academy” (Gubrium et al., 2016, p. 13).

Mapping methods include participatory GIS mapping, asset mapping, food mapping, and other techniques that engage the public in visual mapping, planning, and decision making related to social and cultural environments/contexts. Participatory mapping has been used in a wide variety of disciplines to frame health, educational, and organizational issues within the context of spatial information (e.g., understanding public safety and community violence, informing the development of place-based interventions, mapping the “hot spots” of disease transmission) and can be a powerful approach to engaging underrepresented communities in learning, decision making, planning, and advocacy (S. R. Graham et al., 2011; Larrain & McCall, 2019; Letsela et al., 2018).

Participatory methods such as Photovoice and Videovoice incorporate photography and/or video into data collection and analysis and can promote self-reflection, dialogue, collaboration, power sharing, and voice among stakeholders who are normally not involved in research (Gubrium & Harper, 2016; Lorenz & Kolb, 2009; Packard, 2008). Photovoice and Videovoice go beyond the written word and have the potential to engage people with varying literacy levels, different languages, ages, and cognitive abilities. Photovoice is a well-known and widely-used participatory visual method that engages community members by utilizing photography to identify community strengths and challenges (Catalani & Minkler, 2010; Wang, 2006; Wang & Burris, 1997). Reflection and dialogue based on the photographs lead to community-identified priorities and ultimately a platform for advocacy and social action.

Participatory methods such as digital storytelling, participatory oral history, and participatory theatre-based methods emphasize the narrative aspect of data collection and interpretation. They encourage personal and social change through the sharing of stories and personal and public narrative. Such methods can be used to prompt community dialogue and engagement in decision-making processes (Aranda & Street, 2001; Harper et al., 2004). A “storied approach to research” provides opportunities for stakeholder participation in research by drawing on lived experiences and diverse perspectives (Goodley & Clough, 2004, p. 331). Digital storytelling is used to capture participants’ lived experiences and engage them in making meaning of that experience (e.g., Carlson et al., 2021; Fiddian-Green et al., 2019).

Participatory art-making methods, including collage, drawing, tapestry, murals, and mandalas, utilize arts-based modalities to evoke diverse expression and tap into the “everyday knowledge,” multiple ways of knowing, and creativity of stakeholders (Swantz, 2008, p. 38). In these participatory art-making methods, stakeholders engage in creating and interpreting art as a form of data collection and analysis (Carter & Ford, 2013; Coemans & Hannes, 2017; Jones & Leavy, 2014; Van der Vaart et al., 2018). For instance, Yuen (2016) used collage as a method of inquiry collaborating with Aboriginal women to explore the meaning of leisure and their experiences of healing, and Dutton et al. (2019) used biographical collage with Inuit women to foster capacity building and as a decolonizing process. Mental health studies on anxiety and depression have also used the creation of mandalas in research (Henderson et al., 2007; Lee, 2018; Palmer et al., 2014).

### Domain: Mobilization

Research methods that we classified in the Mobilization domain are those that mobilize action in participatory research by providing tools for decision making, action planning, translation, policy change, and dissemination. All methods categorized in this section provide a specific framework for mobilization that reflects the perspectives and priorities of diverse stakeholders. This domain includes Boot Camp Translation, which aims to promote the translation of evidence-based practices by creating community-focused messages and communication tools. Methods focused on decision making included deliberative methods and the Delphi Process, which bring together diverse stakeholders to reach consensus on important health-related issues. These methods can be used to identify and prioritize health spending, research priorities, and other normative decisions (Burchardt, 2014).

*Boot Camp Translation (BCT)*, developed by the High Plains Research Network (HPRN), is a participatory process to “translate medical information and clinical guidelines into concepts and messages that are understandable, meaningful, and engaging to community members” (Zittleman et al., 2021, p. 339). Working together using a CBPR approach and facilitation team model, community members, researchers, and health experts learn about the health issue and create new paths for community engagement, with an emphasis on creating culturally-relevant messages and changing the local conversation. BCT has been used to address a variety of health issues (e.g., colon cancer screening, asthma, diabetes self-management, high blood pressure, and opioid use disorder and treatment) (Allison et al., 2014; Zittleman et al., 2021). By digging into patient perspectives, the BCT process helps create communication that resonates with communities. For example, in a patient-centered medical homes project, BCT participants engaged in appreciative inquiry to better understand aspects that are salient to patients and tailored messages around those components (Allison et al., 2014). Generally, the BCT process takes 4 to 12 months, including education for participants on the health topic, brainstorming, developing key messages, and dissemination planning (Norman et al., 2013).

A variety of participatory methods fall into the category of consensus approaches that bring diverse stakeholders together to deliberate and identify priorities. Deliberative methods promote informed discussion and consideration of different points of view on specific topics, leading to a decision or recommendation acceptable to all participants. Deliberation as research, Burchardt (2014) points out, generally aims to obtain the informed and considered judgements of participants through a process of public reasoning, with information provided to participants and an expectation that participants’ beliefs and values may change as a result of the deliberation exercise.

Deliberative participatory methods include citizens’ juries, consensus conferences, and citizens’ panels (Abelson et al., 2003). The *Deliberative Democracy Forum* (DDF), developed by the Kettering Foundation, is one example. Procedures for conducting a DDF include several key steps such as framing sessions, facilitated forums (including deliberation), and identifying actions. Cheney et al. (2021) describe a project using DDF to identify community priorities and engage Latinx communities in Riverside, California. After a series of in-home meetings to identify community concerns, stakeholders participated in framing sessions to sort and categorize the community-identified issues. The four final categories that emerged from this process were developed into an Issue Book to help guide the deliberation step. Deliberation was held in four 90-minute forums with community members. Through the forums, community participants were able to reach a consensus on which topic had the most importance.

The *Delphi process* is another consensus method that is often used to elicit and prioritize stakeholder ideas for research-related issues (e.g., research agendas and questions, outcomes). The process involves repeated questioning of selected experts—generally through several rounds of surveys (Hasson et al., 2000). Unlike many participatory methods, Delphi participants may provide their input anonymously via questionnaires, but in-person consensus meetings and workshops are also common. Although the Delphi process has been in use since the 1950s, it is more recently used in participatory research with stakeholders such as patients and service users and incorporated into participatory frameworks such as community-based participatory research or participatory action research. Used in participatory research, stakeholders may be involved beyond answering questionnaires, including collaboration on design and analysis aspects of the research. Fletcher and Marchildon (2014) discuss the Delphi Method’s use in Participatory Action Research (PAR) and the impact of anonymous participation on reducing power differentials among participants. Kezar and Maxey (2016) describe how the Delphi Method can be used in participatory research and introduce the idea of a *change-oriented Delphi*. The Delphi Process has been used in participatory research to elicit consensus on a wide variety of topics, such as research priorities for mental health (Owens et al., 2008), cerebral palsy (Gross et al., 2018), and self-management strategies for bipolar disorder (Michalak et al., 2016). Khodyakov et al. (2017) used a modified online Delphi process to gain patients’ and professionals’ consensus on priorities for comparative effectiveness research on three health conditions. They assessed participants’ experiences using the online process and found that participants were willing to use the process again, felt it was easy to use, and had positive online discussion experiences.

### Domain: Evaluation

Research methods in the Evaluation domain include participatory methods for evaluating partnerships and project processes or outcomes with stakeholders and program participants as active collaborators. Although participatory evaluation can be useful in many contexts, it is a particularly important method for assessing participatory research projects. It provides continuity with the core processes and values that undergird these projects and is well suited for assessing how project outcomes are linked to participatory processes. This is especially important because the distinctive values and processes of participatory research can create challenges for evaluators. For example, these processes increase the complexity inherent in identifying and measuring outcomes, which may occur more gradually than traditional health intervention projects and may include non-traditional outcomes (e.g., community empowerment) (Springett, 2017). Participatory evaluation, therefore, requires a distinct approach to method and metrics, with stakeholder collaboration throughout. The Center for Community Health and Development (n.d.) also emphasizes the importance of making participatory evaluation part of a project from the beginning, so that “beneficiaries become the copilots of a project, making sure that their real needs and those of the community are recognized and addressed.”

Participatory evaluation approaches vary on key dimensions such as who is engaged, how, and for what purpose. Cousins and Whitmore (1998) distinguished between participatory evaluation that supports practical decision making and problem solving (practical participatory evaluation) and that which focuses on empowerment of those who are less powerful or oppressed (transformative participatory evaluation). Our table groups different approaches together under the label participatory evaluation, but we acknowledge that there are many different types and that evaluation teams have many options regarding approach, project design, governance, engagement, and data collection. For example, Fetterman (2019) distinguishes between collaborative, participatory, and empowerment evaluation approaches, based on who is in control of the evaluation process and the role of the evaluator. Chouinard and Milley (2018) reviewed 51 reports describing international participatory evaluation projects. They report that governance arrangements often took the form of stakeholder inclusion in steering committees, research teams, design workshops, task forces and committees, consultation, and participatory planning processes. In addition to these governance arrangements, collaborative evaluation work often occurred through events and meetings, such as workshops, trainings, and other events focused on project activities such as identifying questions, building stakeholder capacity, and interpreting data.

*Partnership evaluation* puts the focus on the process and outcomes of community-based participatory research by assessing partnership functioning and ability to meet objectives (Detroit Community-Academic Urban Research Center, n.d.). The Community-Based Participatory Research Conceptual Model developed by the University of New Mexico Center for Participatory Research (UNM-CPR) and University of Washington’s Indigenous Wellness Research Institute (UWIWRI) provides a conceptual evaluation model that focuses on contexts, group dynamics, research and intervention designs, and outcomes. The model provides specific constructs within each of these levels, but also posits the processes and practices that may influence CBPR outcomes (Wallerstein et al., 2008). Oetzel et al. (2015) validated the psychometric properties of 22 measures related to the CBPR model, including proximal, intermediate, and distal outcome measures. The model was tested by a sample of CBPR partnerships and participants verified and expanded upon previous concepts (Belone et al., 2016), resulting in an evaluation framework that highlights important influences at different levels of the socio-ecological model (e.g., individual, group, community) and by different actors (e.g., community and university). Important factors include socio-economic, cultural, and historical contexts; community capacity and university readiness; trust; reflection; power relations; and mutual learning (Roura et al., 2021). A study of 294 participatory research projects examined the relationship between contextual and partnership practices and partnership outcomes, identifying specific practices that were associated with project outcomes (Duran et al., 2019). The conceptual model and a repository of evaluation tools are available at the Engage for Equity website (Engage for Equity, 2021). An example of application of the model is provided by the Rochester Healthy Community Partnership (RHCP). RHCP’s evaluation process moved through steps of: 1) development of a partnership timeline; 2) facilitated discussion of the CBPR conceptual model to discuss partnership constructs and consider how the constructs influenced the partnership’s work; 3) interviews and surveys using adapted instruments guided by the CBPR conceptual model; and 4) participatory data analysis (Reese et al., 2019).

*Ripple Effects Mapping (REM)* is a retrospective, qualitative evaluation approach that brings together project stakeholders to map the chain of effects of a program or collaboration in complex, real-life settings (Chazdon et al., 2017). Four essential components of REM include Appreciative Inquiry, a participatory approach, interactive group interviewing and reflection, and radiant thinking (Mind Mapping) (Chazdon & Langan, 2017). Evaluations often incorporate the Community Capitals Framework (CCF) and employ approaches such as “web mapping” and “in-depth rippling.” REM has been used in a variety of fields. Welborn et al. (2016) used it in six project sites to assess a civic dialog process that engaged residents to explore poverty in their community and act on identified concerns. They concluded that the process was easy for participants to understand and provided real-time deliberation about responses. They also found that the process helped participants recognize and appreciate program impacts, spurring new energy among the groups. Washburn et al. (2020) used REM to evaluate a health education program and capture program impacts beyond participant behavior change to identify community-level ripple effects. Others have used similar approaches to capture ripple effects of participatory research (Dias et al., 2021; Zimmerman et al., 2019).

## Discussion

Despite its positive contributions to research and partnership, conducting PHR also entails many challenges. Since a community is not a purely geopolitical nor homogeneous entity, collaboratively defining who comprises the community and its stakeholders is a critical first step that has consequences throughout the project (MacQueen et al., 2001; Roura et al., 2021). Although the active participation of community partners and stakeholders is essential to PHR, managing power relations, conflicts of interests, and diverse perspectives requires attention, time, and continuous dialogue and negotiation efforts throughout the research process to ensure equitable involvement of all partners (both academic and non-academic) (Dias et al., 2018; Newman et al., 2011). Additionally, conducting participatory research involves working closely with non-academic partners with different backgrounds and competencies regarding research activities, such as data collection and analysis. In practice, it is often difficult to ensure equitable involvement of different partners throughout project activities (Dias & Gama, 2014; Ramsden et al., 2010). Differences in competencies, commitment, interests, motivations, expectations, and the need for continued adjustments to the project add to the necessary commitment of time and resources (Abma et al., 2019).

Using research methods developed or adapted for stakeholder engagement and collaborative research does not guarantee that these challenges will be reduced or eliminated, but should provide the foundation needed to plan for and implement strategies to address these issues. We have highlighted a range of methods for: 1) collaborative participation in the processes of engaging stakeholders and building capacity; 2) facilitating group processes for exploration, dialogue, priority setting, and question development; 3) sharing and co-production of stakeholder experiences and ideas through alternative, multimodal approaches to data collection and interpretation; 4) mobilizing for decision making, action planning, translation, policy change, and dissemination; and 5) evaluating participatory research. Researchers and their partners must evaluate the suitability of PHR methods for their project goals and purposes based on available resources (e.g., time, expertise, funding) and assess relevant tradeoffs. For example, while more time consuming, longer-term engagement may help build trust and capacity and is critical to achieving co-production of research. However, shorter-term engagement may be a better fit for some projects or a starting point for those new to PHR.

### Limitations

This article was prepared largely for other researchers seeking to familiarize themselves with a range of PHR methods and did not involve collaboration with different types of stakeholders; however, we welcome feedback from all stakeholders as well as suggestions related to methods that were not covered here. This overview contains examples in each of the five domains presented and does not aim to provide an exhaustive list of relevant methods. In addition, we decided to limit the scope to methods and have not included the many useful tools that are available for PHR. Beyond noting some of the strengths and challenges related to each method that have been reported in the literature, we did not evaluate the effectiveness of the methods, nor did we discuss ethical challenges associated with the methods. Our categorization of methods into the five domains is meant to help readers match partnership and project goals with available methods, but we fully acknowledge that the methods presented here are useful in multiple contexts and to accomplish a variety of goals.

## Conclusion and Implications for Future Research

Research methods can be effectively implemented in participatory ways. It is key to nurture a collaborative environment that embodies PHR principles, such as the active engagement of stakeholders and communities in the conceptualization of research questions and in the choice of methodological approaches. The promotion of all partners’ engagement encourages openness and opportunity for continuous dialogue, exchange of knowledge, respect and trust, and self-reflexivity. Echoing the words of Wallerstein (2020), “the commitment to practice participatory methods is critically important to ensure genuine engagement” as we collectively work towards achieving health and social justice and eliminating inequities. Opportunities for future research include process and outcome evaluation of participatory health research using established and emerging participatory research methods.

## Figures and Tables

**Table 1.** Engagement and Capacity Building

Engagement and capacity building. This domain includes methods that draw stakeholders into community-engaged research at the initial planning stages, periodically at key points in the project, and on an ongoing basis through the formation of collaborative working and infrastructure support. It also includes strategies for capacity building, with the aim of supporting people to continue to engage with the research.					
	Type/Brief Description	Goals	Participants	Strengths	Challenges
**Community engagement studio** (Joosten et al., 2015, 2018, 2021)	Consultative community review of research	-Project-specific community input is used to enhance the design, implementation, and dissemination of research -Assessing and improving ethics, relevance, and appropriateness of research	-Community residents -Members of the population the research is intended to benefit -Academic researchers	-Feedback from underrepresented and specialized populations -Useful at various stages of the research process	-Requires institutional support -Stakeholders lack decision-making power
**CBPR Charrette** (Samuel et al., 2018; Smith et al., 2020) See also https://www.involve.org.uk/resources/methods/design-charrettes	A collaborative planning process to assist with partnership development, stakeholder engagement, and decision-making infrastructure	-Provide community and academic research partners with technical assistance -Provide an interactive forum in order to clarify problems that people want to address -Identify needs for different types of knowledge and expertise -Find sources of expertise and technical assistance to support partnership development, engagement, and decision-making	-Community groups and local citizens -Academic researchers -The type of stakeholders depends on the nature of the problem(s) to be addressed	-Tailored, time-limited sessions -Identifies issues and possible solutions -Periodic small-group work informs the larger process -Creates positive collaboration across diverse stakeholders -Reviews feasibility and relevance of possible projects -Produces realistic visions of what can be done and -Co-designed, detailed plan to guide next steps for the partnership	-Participants may not be representative of the wider community -Time compression: Intensive sessions over a short time period may exclude people who cannot commit the time -The logistics for planning the sessions require adequate administrative resources -Potential costs -Involvement of diverse people over a short time period requires skilled community and academic co-facilitators, to ensure participation is not dominated by experts -Transparent communication about the process is needed -May create false expectations
**Patient-research networks (PRNs)** (Chalmers et al., 2013; Dean et al., 2021; Marschhauser et al., 2021; Nowell et al., 2018)	Networks that bring together patient groups focusing on specific health conditions to set research priorities and contribute to patient-centered outcomes research	-Bring together health data and patient partnerships to enable large-scale patient-centered clinical research Use networks to: -Create patient-centered research agendas -Identify patient-valued outcomes -Design patient-centered research	-Patients and caregivers interested in sharing their health information and participating in research -Researchers	Participant governance helps to: -Prioritize research questions -Enhance research design, including diverse and representative enrollment, data sharing and ethics -Sustain and expand networks -Identify effective approaches to disseminating results	-Conflict and lack of agreement across different patient groups in the network -Skilled facilitation is needed to manage issues of imbalance of power, to develop trust (Abma et al., 2019)
**CBPR community advisory board (CAB)** (Dias et al., 2018; Keygnaert et al., 2015; Newman et al., 2011)	Collaborative, ongoing leadership for CBPR	-Facilitate community voice in research -Provide feedback on research processes -Identify community needs, interests, and research priorities -Provide ethics oversight	Community advisory board members are typically chosen from the community of interest, e.g. -Community residents -Organizational representatives -Underrepresented groups -People who can access the resources and skills needed	-Improve buy-in, representation, quality, and effectiveness of research -Increase capacity for communities to resolve problems via ongoing training and technical support -Opportunities to translate research into action	-Time consuming and labor intensive -May not be representative of the communities involved -CABs must clarify (often shifting) roles with academic researchers, which may range from a limited advisory role, to collaboration, to active control and oversight of the research project -Differing priorities across community and academic partners -Differing priorities between CAB and research funders

**Table 2.** Exploration and Visioning

Exploration and Visioning. Methods in this domain supports various stages of research development, such as community dialogue, stakeholder priority setting, developing research questions, and exploring the meaning, causes, or solutions to specific problems.					
	Type/Brief Description	Goals	Participants	Strengths	Challenges
**SEED Method** (Rafie et al., 2019; Zimmerman et al., 2020; Zimmerman & Cook, 2021)	Multi-stakeholder approach to research development	-Explore issues from stakeholder perspective -Develop and prioritize research questions or action strategies -Action planning	-Community members -Underrepresented populations -Patients -Caregivers -Stakeholders -Researchers -Multi-stakeholder	-Identifying and recruiting specific subgroups of stakeholders -Focus on participant expertise and collaboration with diverse participants -Can build capacity among participants -Structured process with tools, instructions, and facilitator guides	-Time and resource commitment -Experience with participatory research is helpful
**Concept mapping** (Burke et al., 2005; Trochim & Kane, 2005)	Mixed methods, visual approach to priority setting/program development	-Action planning -Program development -Capacity building -Priority setting	-Community members -Relevant stakeholders	-Visual representation and clustering of large number of ideas -Flexibility as to how participatory or community-engaged the process is -Structured process with guidelines to follow	-Sorting and rating task can be tedious and burdensome -Requires skilled statistician and specialized software to conduct analysis step
**Community forum (also known as public forums, listening sessions, town hall meetings)** (Becker et al., 2003; Monroe et al., 2009)	Large group meeting to promote dialogue, problem solving, and collaboration	-Platform for community dialogue -Broaden public understanding -Facilitate coordination across multiple systems and stakeholders	-Community members	-Allows large groups of people to exchange ideas -Engages public in research and problem solving	-Requires skilled facilitator -Space requirements -Requires scheduling and logistical support
Future search (Weisbord & Janoff, 2000, 2010)	Large group planning process	-Bring diverse stakeholders together to plan their common desired future, choose priorities, and move to action -Strategic planning	-Stakeholders	-Brings large groups of diverse stakeholders together -Gets “whole systems” together in the room -Consider the big picture before moving to action	-Lengthy (usually 2 ½ to 3 days) -Requires skilled facilitator who can manage large groups -Requires scheduling and logistical support
**World café** (Fouché & Light, 2011; Jorgenson & Steier, 2013)	Collaborative approach to foster dialogue	-Conversational approach to engagement and dialogue around a critical question -Generating ideas, sharing knowledge, stimulating innovative thinking, and exploring actions - Develop strategies or research questions to address a community concern	-Community members -Stakeholders	-Assumes that knowledge and wisdom needed are already present and accessible and that solutions appear when people come together and get creative - “Café” setting provides an intimate, comfortable atmosphere -Fosters creative and collaborative solution-finding -Diverse perspectives -All participants have a chance to be heard; speak from the heart, rather than lecturing or taking a stand	-Some participants may resist an unconventional approach
**Group level assessment** (Vaughn & DeJonckheere, 2019; Vaughn & Lohmueller, 2014)	Large group planning process	-Idea generation -Needs assessment and prioritization -Action planning	-Community members -Relevant stakeholders	-Can build community capacity -Supports non-researcher voices and collaboration in research process -Links research to action -Large group (15–60 stakeholders per session) -Structured format of 7 steps -Allows different status stakeholders to interact and gain perspectives	-Requires skilled facilitator who can manage large groups -Space requirements -Requires scheduling and logistical support -Potential for inequitable power dynamics among different status stakeholders

**Table 3.** Visual and Narrative

Visual and Narrative. This domain includes participatory visual and narrative approaches to data collection, analysis, and interpretation.					
	Type/Brief Description	Goals	Participants	Strengths	Challenges
**Participatory GIS mapping** (Brown et al., 2014; Elwood, 2006; Rouse et al., 2007) **Asset mapping** (Kramer et al., 2012; Lightfoot et al., 2014; Mosavel et al., 2018) **Food mapping** (Jacobi et al., 2019; Sweeney et al., 2016)	Spatial data method	-Generate spatially explicit information for multiple decision-making purposes -Disease surveillance	-Stakeholders	-Creates a new perspective on research for local stakeholders -Visual aspect is easily engaging -Adaptable for different social and cultural environments	-Can be difficult to use -Requires specific technical skills -Can be expensive and time consuming
**Photovoice** (Catalani & Minkler, 2010; Wang et al., 1998; Wang & Burris, 1997) **Videovoice** (Catalani et al., 2012; Li et al., 2019; Warren et al., 2014)	Visual, arts-based, small group method	-Promotes social action through photography so that participants can document their lives and communities	-Community members -Specialized populations (e.g., youth)	-Works well regardless of language/literacy -Flexible -Accessible	-Logistical support -Ongoing participation required
**Storytelling (Digital)** (de Jager et al., 2017; Rieger et al., 2018) —also oral histories; theatre-based	Arts-based, qualitative research method	-Create short videos that capture and share participants’ lived experiences as counter-narratives	-Social inequity groups -Vulnerable and marginalized populations	-Participatory approach to making meaning, engaging in decision making, active involvement in research process -Suited to knowledge translation and dissemination	-Limited publication of studies -Stories are usually very brief (3–5 minutes) and may not capture potentially important content and context -Requires skilled researchers to minimize bias, influence, etc. -Not always part of a participatory process
**Participatory art-making methods** (Carter & Ford, 2013; Coemans & Hannes, 2017; Jones & Leavy, 2014; Van der Vaart et al., 2018) —examples: collage, drawing, tapestry, murals, mandalas	Methods that use art/visual methods to create data	-Provides opportunity for creative expression beyond words -Understand and represent human experience and phenomena of interest	-Community members -Specialized populations (e.g., youth)	-Fun, creative -Engaging -Works well regardless of language/literacy -Taps into lived experience and feelings	-Can be intimidating if the method is perceived to require artistic abilities -Considered by some to be too subjective and non-rigorous

**Table 4.** Mobilization

Mobilization. This domain includes methods that mobilize action in participatory research by providing tools for decision making, action planning, translation, policy change, and dissemination.					
	Type/Brief Description	Goals	Participants	Strengths	Challenges
**Boot camp translation** (Allison et al., 2014; Norman et al., 2013; Zittleman et al., 2021)	Collaborative process for community-based teams	-Health promotion -Evidence-to-practice	-Community members -Multi-stakeholder	-Community-focused messages and communication tools -Training program available	-Time and funding resource needs
**Deliberative methods** (Cheney et al., 2018, 2021)	Consensus process for discussion, decision-making, and mobilizing action	-Engage stakeholders in discussion and obtain informed public input on competing solutions -Participants consider the pros and cons of each choice and then reach consensus -Setting priorities for research or spending	-Community members -Stakeholders	-Community-driven approach to addressing issues -Brings together diverse participants	-Requires a lot of preparation
**Delphi processes** (Fletcher & Marchildon, 2014; Kezar & Maxey, 2016)	Consensus method	-Improve understanding of problems, opportunities, and solutions -Systematically collect opinions from experts and stakeholders -Usually conducted through anonymous surveys, in several rounds	-Stakeholders -Experts -Researchers	-Rapid consensus can be achieved -Anonymous -Can include a wide range of experts -Relatively low cost to administer and analyze -Participants can be from a wide geographic area -Can be done online -Flexible number of participants	-Success of the method depends on the selection, expertise, and motivation of participants -Attrition between rounds

**Table 5.** Evaluation

Evaluation. This domain includes participatory methods for evaluating project processes or outcomes.					
	Type/Brief Description	Goals	Participants	Strengths	Challenges
**Participatory evaluation** (Center for Community Health and Development, n.d.; Chouinard & Milley, 2018; Cousins & Whitmore, 1998; Dias et al., 2021)	Encompasses a number of participatory approaches to evaluating programs (e.g., participatory evaluation, collaborative evaluation, empowerment evaluation)	-Address inequities in evaluation practice -Align programs with community needs -Increase ownership of the evaluation process and results	-Program participants -Program staff -Partners -Sponsors -Evaluator -Or all those with a stake in the outcome	-Inclusive -Many ways to design studies and collect data - Focus on capacity building and empowerment - Good fit for participatory projects	-Some approaches do not distinguish clearly between participants as collaborators or as data sources -May take longer than traditional evaluation
**Partnership evaluation** (Belone et al., 2016; Oetzel et al., 2015; Wallerstein et al., 2008)	Evaluation of CBPR partnership practices and outcomes	-Use conceptual/logic model of CBPR partnership processes	-CBPR team members and partners	-Captures many nuances of CBPR processes and outcomes -Availability of scales and instruments -Measures have been tested for validity	-Complex model -Can be labor intensive to implement as evaluation approach
**Ripple effects mapping** (Chazdon et al., 2017)	Participatory method to retrospectively and visually map the chain of effects resulting from a program or collaboration	-Reflect on and document intended and unintended effects -Use the Community Capitals Framework -Evaluate longitudinal impacts	-Program participants -Program staff/board members -Coalition members -Community members -Stakeholders	-Uncover effects that may be missed by traditional evaluation (intended and unintended)	Can be difficult to: -Get timing right -Decide who to include -Achieve consistency across sites -Show that reported impacts are attributable to the program
